# Managing birds of conservation concern on sandy shores: How much room for future conservation actions is there?

**DOI:** 10.1002/ece3.4564

**Published:** 2018-10-17

**Authors:** Brooke Maslo, Karen Leu, Todd Pover, Michael A. Weston, Thomas A. Schlacher

**Affiliations:** ^1^ Ecology, Evolution, and Natural Resources Rutgers, The State University of New Jersey New Brunswick New Jersey; ^2^ Rutgers Cooperative Extension, New Jersey Agricultural Experiment Station Rutgers, The State University of New Jersey New Brunswick New Jersey; ^3^ Conserve Wildlife Foundation of New Jersey Trenton New Jersey; ^4^ Centre for Integrative Ecology School of Life and Environmental Sciences, Deakin University Burwood Victoria Australia; ^5^ The ANIMAL Research Centre: Health + Ecology + Conservation University of the Sunshine Coast Maroochydore Queensland Australia; ^6^ School of Science and Engineering, University of the Sunshine Coast Maroochydore Queensland Australia

**Keywords:** beach‐nesting birds, conservation planning, habitat protection, species distribution modeling, wildlife management

## Abstract

Resource limitations often prevent the active management required to maintain habitat quality in protected areas. Because restrictions in access or allowable public activities are the sole conservation measure in these locations, an important question to consider is whether species of conservation concern truly benefit from parcels that are shielded from human disturbance. Here, we assess the conservation benefit of protecting birds from human recreation on over 204 km of sandy beaches by (a) estimating the total area of beach‐nesting bird habitat that has been created by conservation protections; (b) quantifying the change in nesting habitat extent should further conservation protections be implemented; and (c) providing data to inform future protected area expansion. We use a maximum entropy species distribution modeling approach to estimate the extent and quality of suitable habitat for four beach‐nesting bird species of conservation concern under the existing management regime and compare it to scenarios in which the entire study area is either unprotected of fully protected from human disturbance. Managing humans has dramatic conservation returns for least terns and piping plovers, creating extensive nesting habitat that otherwise would not exist. There is considerable scope for conservation gains, potentially tripling the extent of nesting areas. Expanding conservation footprints for American oystercatchers and black skimmers is predicted to enhance the quality of existing nesting areas. The work demonstrates the utility of modeling changes in habitat suitability to inform protected area expansion on ocean beaches and coastal dunes.

## 1 INTRODUCTION

If one cannot catch a bird of paradise, better take a wet hen Nikita Khrushchev

Habitat loss and declines in environmental quality are widely recognized as pivotal threats to biodiversity and wildlife populations; they are targeted by many conservation actions worldwide (Meir, Andelman, & Possingham, 2004). To mitigate these threats, conservation planning often involves strategically protecting a network of reserves to promote recruitment of individuals (Gell & Roberts, 2003), improve connectivity among habitat fragments (Engelhard et al., 2017; Possingham, Ball, & Andelman, 2000; Williams, ReVelle, & Levin, 2004), and protect target species from deleterious anthropogenic processes (i.e., disturbance, exploitation, contamination). However, protected areas may not effectively conserve target populations for several reasons (Althaus, Williams, Alderslade, & Schlacher, 2017; Gilby et al., 2017; Huijbers et al., 2015). Among them, existing protected areas may be in locations that do not significantly benefit target populations (Gilby et al., 2017), instead reflecting historic human settlement patterns or exhibiting low commercial or high recreational and scenic values (Joppa & Pfaff, 2009; Scott et al., 2001). Alternatively, resource limitations often prevent the active management required to maintain habitat quality across both the site and network scale (Arponen, 2012; Murdoch et al., 2007). Therefore, on‐the‐ground protected areas are often a collection of ad hoc or opportunistic land acquisitions that are protected from consumptive anthropogenic activities, but that are not always actively managed to improve habitat quality (Barr, Watson, Possingham, Iwamura, & Fuller, 2016; Maslo, Lockwood, & Leu, 2015). Given that these areas receive no active conservation intervention other than restrictions in access or allowable public activities, an important question to consider is whether species of conservation concern truly benefit from parcels that are shielded from human disturbance. If they do, then protecting additional sites may increase the scope of conservation outcomes for species of concern.

The impacts of human presence on wildlife species are well documented, with the clearest links occurring between consumptive anthropogenic activities (i.e., harvesting species) and species’ survival and reproduction. A substantial body of literature highlighting the potential impacts of non‐lethal activities (i.e., ecotourism, hiking) also exists (e.g., Claudet & Fraschetti, 2010, Murphy & Romanuk, 2014). However, quantifying the latter impacts within a conservation management context remains a significant challenge (Weston, Schlacher, & Lynn, 2014). Doing so could provide meaningful benchmarks for at least three overarching conservation questions commonly asked by wildlife managers: (a) Is the current level of protection enough to meet conservation goals? (b) Is there scope for conservation expansion (i.e., how much habitat can be added through human disturbance protections)? and (c) Where may human disturbance protections increase habitat quality enough to benefit target species? Here, we address these questions by using a species distribution modeling approach to test how protection influences breeding habitat suitability for four beach‐nesting birds of conservation concern along the eastern Atlantic coastline of the United States.

Suitable breeding habitat for beach‐nesting birds generally consists of sparsely vegetated, gently sloping sandy substrates in close proximity to intertidal or nearshore marine foraging grounds (Burger & Gochfeld, 1990; Gochfeld, 1978; Maslo, Handel, & Pover, 2011; McGowan, Simons, Golder, & Cordes, 2005). Because many coastal areas are densely populated by humans (Lockwood & Maslo, 2014), beach‐nesting birds are severely threatened by both direct and indirect anthropogenic impacts that degrade habitat quality (Defeo et al., 2009; Lima, 2009; Schlacher, Lucrezi, et al., 2016). Population growth is limited by poor reproductive success stemming from predation, flooding, and human disturbance (Van De Pol et al., 2010, Cohen et al., 2016, Maslo, Schlacher, et al., 2016). This scenario thus provides an ideal system in which to test the impact of human disturbance protections on a conservation reserve network.

Here, we model how protections from human disturbance modify the size and distribution of habitat that will likely support nesting by beach‐nesting birds of conservation concern. We specifically ask three complementary questions: (a) *Does protecting habitat from human disturbance increase its quality? *(b)* How much habitat is protected relative to what potentially exists?* and (c)* What is the potential future conservation benefit if protection from recreational human activities were to be extended to all potential nesting habitat along the coastline*? We address these questions along the densely developed coastline of New Jersey (USA) to examine whether and how any of the above effects are species‐specific.

## 2 METHODS

### 2.1 Target species and study area

**Figure 1:**
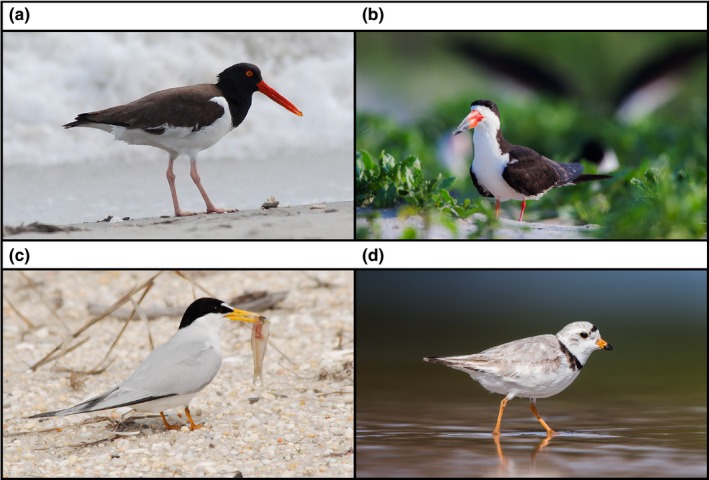
Focal beach‐nesting birds of conservation concern in New Jersey, USA, include the following: (a) American oystercatcher (*Haemotopus palliatus*); (b) black skimmer (*Rynchops niger*); (c) least tern (*Sterna antillarum*); and (d) piping plover (*Charadrius melodus*). Photograph credit: Bill Lynch (American oystercatcher, least tern); Northside Jim (black skimmer, piping plover)

The target species are four beach‐nesting birds of conservation concern in eastern North America: American oystercatcher (*Haemotopus palliatus*), black skimmer (*Rynchops niger*), least tern (*Sterna antillarum*), and piping plover (*Charadrius melodus*; Figure 1). Black skimmers and least terns are colonial nesters (Brunton, 1999; Erwin, Galli, & Burger, 1981), while American oystercatchers and piping plovers nest as solitary pairs (Maslo et al., 2011; Wilke et al., 2009). Breeding sites for all species are typically found in areas of low elevation with gently sloping, low‐lying dunes (Gochfeld, 1978, 1983 ; Maslo et al., 2011; McGowan et al., 2005). Nests are positioned between the spring high tide mark and the seaward toe of the dune line; these locations offer both protection from storm tides, as well as lowered detection probability by avian and mammalian predators (Mazzocchi & Forys, 2005; McGowan et al., 2005). Terns and skimmers feed their chicks, which generally remain within the colony limits. Piping plover chicks are precocial, meaning that they are mobile and able to feed themselves within hours of hatching (Melvin, Griffin, & Macivor, 1991). Although oystercatcher chicks rely on their parents for food, they do not remain at the nest‐site during the pre‐fledging stage (AMOY, 2014). Availability of, and unrestricted access to, foraging areas (i.e., intertidal zone, wrack line, tidal ponds) are critical for these individuals to reach fledgling stage successfully (Loegering & Fraser, 1995; Sabine, Meyers, Moore, & Schweitzer, 2008).

**Figure 2:**
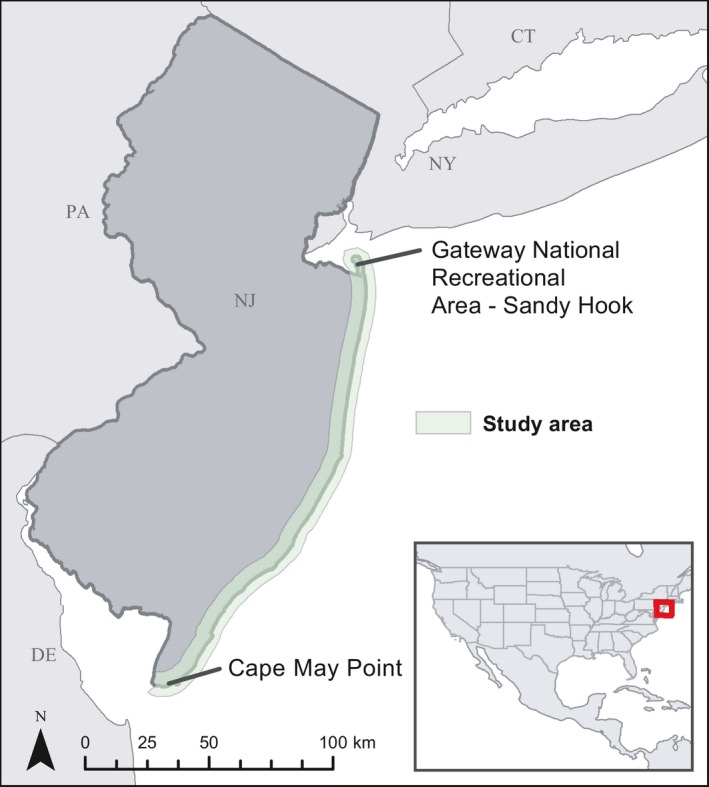
The 1,040 km^2^ study area included all land <5 km of the New Jersey, US coastline, extending from Gateway National Recreation Area – Sandy Hook Unit in the North to Cape May Point in the South

The study area encompasses all land within 5 km of the New Jersey, US coastline (NJDEP, 2007), between Gateway National Recreation Area – Sandy Hook Unit and Cape May Point (~1,040 km^2^; Figure 2) and includes all potential nesting habitat (specifically beaches, dunes, salt marsh, and tidal flats) of our target species. Much of the landscape within the study boundary (754 km^2^) has been heavily altered for human use (urban, residential lands, etc.), leaving approximately 286 km^2^ potentially available for beach‐nesting birds. The human population along coastal New Jersey is dense at 525 persons/km^2^ (NOAA, 2013) and increases substantially during the summer months. Approximately 35% of New Jersey's beach‐nesting bird pairs occur on federally protected wildlife refuges that are closed to the public during the nesting season (Heiser & Davis, 2017). The remaining pairs nest on publicly owned lands with a primary focus on recreation, including sunbathing, action sports (surfing, kite‐surfing, etc.), fishing, campfires, and off‐road driving. In these locations, smaller nesting areas are maintained through beach management cooperative agreements among local municipalities/site owners, the United States Fish and Wildlife Service (USFWS), and the New Jersey Department of Environmental Protection. These agreements are intended to provide long‐term protection and recovery of federally or state listed species, while balancing the need for recreational use and storm protection. Designated protected areas are a required “term and condition” as part of the provisions of the U.S. Endangered Species Act (Congress, 1973) and are determined through various habitat assessments conducted by the USFWS (2002, 2016). Representatives from the State's Endangered and Nongame Species Program and the USFWS meet with local land managers to develop and draft the beach management plans. Upon completion, the plans are approved through resolution by the local governing body (i.e., Borough/City Council), after which a Memorandum of Agreement is signed by all parties.

In unprotected areas, human activities are typically intensive during the summer (late May through early September) but also do occur throughout the entire year. Pedestrian and vehicular traffic are permitted between the high tide line and the seaward toe of the primary dune. In areas under conservation protections, pedestrian and vehicular access are restricted in nesting and foraging areas for all or part of the year (through closures and symbolic fencing). Other restrictions include prohibition of beach‐raking, dogs, kite‐flying, fireworks, and other recreational activities.

### 2.2 Modeling nesting habitat suitability for beach‐nesting birds

Maslo, Leu, et al. (2016) predicted the distribution of each species using a spatially explicit maximum entropy modeling approach. Briefly, they trained models using nest or nesting colony (depending upon the species) occurrence data for the years 2007–2011 (*N* = 1,288) obtained from the New Jersey Endangered and Nongame Species Program. They tested eight predictor variables in the models, which included factors representing the physical characteristics of the landscape, behavior of the birds, and the intensity of recreational activities. Environmental predictors included land cover (e.g., beach, vegetated dune community, marsh), elevation, slope, and distance to both the high tide line and non‐ocean tidal waters. They included beach width (narrow vs. wide shorelines), as well as adequate breeding territory size, by calculating the total area of sandy beach and marsh (separately) within 100 m.

They also included differences among habitats with respect to conservation status by classifying them into four management zones of increasing protection status: (a) unprotected areas (~27,490 ha); (b) species precautionary areas (~230 ha); (c) species protection areas (~565 ha); and (d) closed areas (~285 ha). In unprotected areas, beaches are maintained for human use either during the summer (late May through early September) or throughout the entire year, and pedestrian access is permitted from the high tide line to the seaward toe of the primary dune. Recreational beach vehicles typically are permitted from October to April, and authorized vehicles (e.g., refuse pick‐up, lifeguards) are permitted all year. Precautionary areas have temporary no‐rake and no‐vehicle designations, but human access is only restricted if birds initiate nests. Species protection areas have more proactive interventions, including full breeding season beach‐rake, vehicle, and dog prohibitions. In addition, anticipated nesting areas are delineated with symbolic fencing. Closed areas have no public access throughout the breeding season. All protected areas are monitored by state or federal wildlife agency personnel during the breeding season. With a few exceptions, signage, symbolic fencing, and agency presence effectively minimize human disturbance of breeding areas. Enforcement typically comes in the form of outreach and education (i.e., friendly conversations between monitors and trespassers). Egregious offenses (i.e., nest or egg destruction) are rare and handled by state conservation officers or the local police authorities.

Results of the models indicated that land cover and distance to the high tide line were important predictors of nesting habitat. American oystercatchers and black skimmers were also influenced by the distance to non‐ocean tidal waters, while least terns and piping plover nesting habitat was more dependent on the beach size and width. Elevation and slope had relatively little predictive power. Importantly, management zone was ranked among the top four predictors of nesting suitability for all species. The models, tested on an independent data set, generated the probability of a nest occurrence for each target species, or *suitability score*, at a 10‐m cell resolution across the study area. They defined suitable habitat as those cells with a suitability score greater than or equal to the calculated 10‐percentile training presence threshold (i.e., the minimum suitability score above which 90% of the occurrence data fall (Maslo, Leu, et al., 2016)). Suitability thresholds for each species were as follows: 0.208 (American oystercatcher); 0.300 (black skimmer); 0.382 (least tern); and 0.474 (piping plover).

### 2.3 Modeling the influence of human disturbance protections on habitat suitability

To assess how protection from most human disturbance influences both the current extent and quality of suitable nesting habitat, as well as the spatial scope of future conservation benefits, we ran the species distribution models under two hypothetical protection scenarios. In scenario (a)—“*unprotected”—*we asked how much suitable nesting habitat would be present if no human disturbance protections existed across the study area. For this model run, we replaced the original management zone layer with one in which the entire study area was designated as unprotected. In scenario (b)—“*all protected”—*we asked how a hypothetical expansion of conservation protections would change the area and quality of habitats that are predicted to support nesting. However, closing off all beaches to human access is an unrealistic management scenario. On the other hand, species precautionary areas (as demonstrated by Maslo, Leu, et al., 2016) offer little conservation benefit relative to unprotected areas. Therefore, we classified the entire study area as a species protection zone, which offers the greatest amount of human disturbance protections without completely closing areas to human access. This scenario effectively explores the upper limit of available habitat for beach‐nesting birds along this coastline.

We fitted models using maximum entropy modeling software using the linear, product and quadratic model parameters (as in Maslo, Leu, et al., 2016) and evaluated the models by using a separate test data file consisting of nest/colony occurrence from the 2012 breeding season. We verified model fit by confirming that the area under the curve (AUC) score and the permutation importance values generated by the analysis were consistent with that of Maslo, Leu, et al. (2016). We then calculated the total extent of suitable habitat available for each species under the current protection regime, and we compared it to the total extent of habitat available under the unprotected and all protected scenarios.

## 3 RESULTS

### 3.1 Managing human disturbance expands habitat for birds

**Figure 3:**
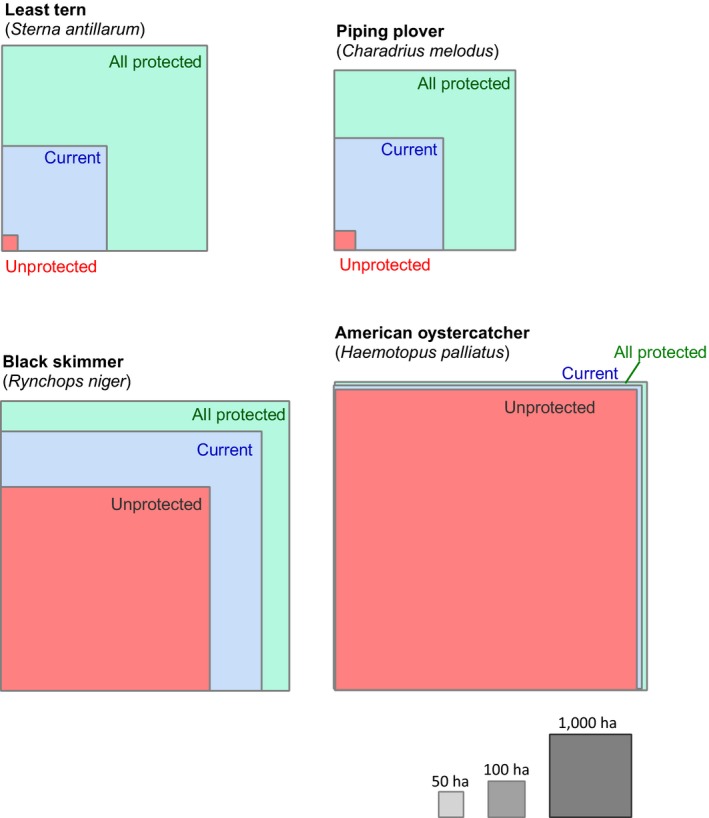
Change in total suitable area for four species of beach‐nesting birds on the New Jersey, US coastline, under three protection scenarios. Colored boxes represent a scaled gradation in protection effort from no active conservation interventions to lower disturbance mainly from recreational activities (pink), the current management regime (blue), and expansion of bird conservation to the entire coastline of New Jersey (green)

Current conservation regulations have dramatically increased the amount of suitable nesting habitat relative to the unprotected scenario. However, total extent of nesting habitat availability varied significantly among species (Table 1; Figure 3). Least terns and piping plovers benefitted most from conservation investment, both species having virtually no available habitat without protections from human disturbance. The current conservation protection network increases least tern and piping plover nesting habitat 211‐fold and 35‐fold, respectively. Black skimmers and American oystercatchers have also benefitted from the current human disturbance protections, but habitat gains relative to the unprotected scenario are more modest (1.6‐fold and 1.03‐fold, respectively). Without human disturbance protections, black skimmer nesting habitat extends across 2,247 ha along the NJ coastline; the current management regime increased that extent to 3,605 ha. Total American oystercatcher nesting habitat increased by only 142 ha (Table 1, Figure 3).

**Table 1:** Total habitat available to threatened beach‐nesting bird species under three different scenarios of varying degrees of protection against human disturbance. Numbers in parentheses indicate the total area of habitat gained relative to the previous protection level. Italicized numbers reflect the *x*‐fold change in suitable habitat relative to the previous protection level

Species	Metric	Unprotected	Current protections	All protected
Least tern	Habitat area	2.8 ha	592 ha	2,266 ha
	Gain in habitat area		(589 ha)	(1,674 ha)
	*x‐*fold change in area		*211.32x*	*3.83x*
Piping plover	Habitat area	18.1 ha	649 ha	1,725 ha
	Gain in habitat (area)		(631 ha)	(1,075 ha)
	*x‐*fold change in area		*35.86x*	*2.66x*
Black skimmer	Habitat area	2,247.2 ha	3,605 ha	4,382 ha
	Gain in habitat area		(1,358 ha)	(776 ha)
	*x‐*fold change in area		*1.60x*	*1.22x*
American oystercatcher	Habitat area	4,778.6 ha	4,920 ha	5,080 ha
	Gain in habitat area		(142 ha)	(160 ha)
	*x‐*fold change in area		*1.03x*	*1.03x*

Expanding conservation protections to the entire study area under a future “all protected” scenario translates into substantial increases in suitable nesting habitats for least terns (3.8‐fold gain from the current 592 to 2,266 ha in the future) and sizeable gains for piping plovers (2.6‐fold gain from 649 to 1724 ha). Full protection is predicted to expand black skimmer habitat by 776 ha (1.22‐fold increase), and a more modest expansion of 160 ha (1.03‐fold increase) for oystercatchers.

**Figure 4:**
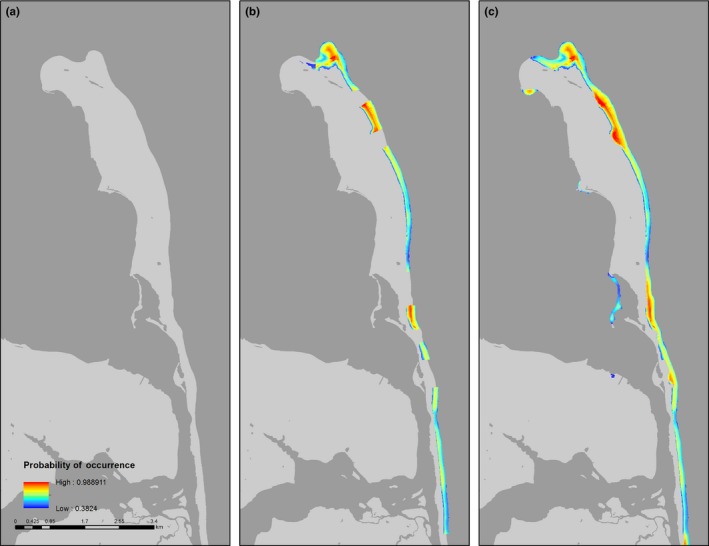
Example map of changes to the location and extent of habitat suitable for nesting by least terns (colored areas) in Gateway National Recreation Area, New Jersey, USA (a) without human disturbance protections, (b) under the current levels of protection, and (c) under a scenario in which all potential habitat is protected from human disturbance. Suitable habitat is defined as 10 x 10 m cells with a probability of nest occurrence above the calculated suitability threshold in blue (0.3824); warmer colors (red, yellow, orange) indicate areas with higher suitability. No nesting habitat exists for least terns without protection from human disturbance. The current protection scenario increases habitat extent considerably, but habitat extent is maximized under full protection

**Figure 5:**
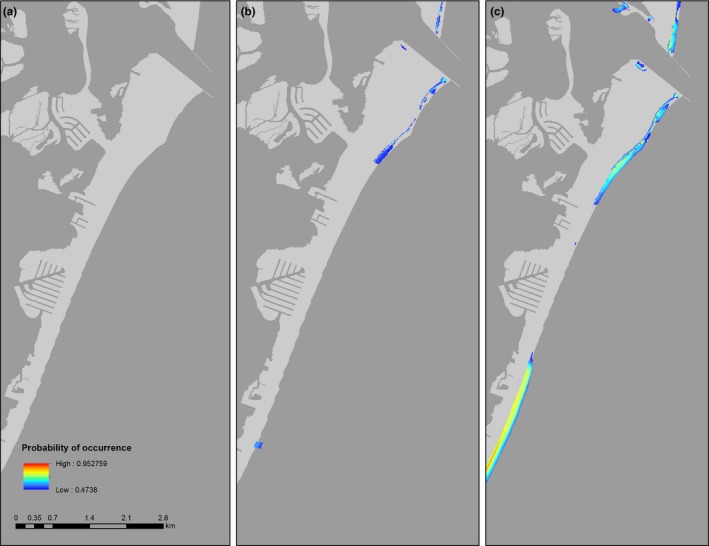
Example map of changes to the location and extent of habitat suitable for nesting by piping plovers (colored areas) on Long Beach Island, New Jersey, USA (a) without human disturbance protections, (b) under the current levels of protection, and (c) under a scenario in which all potential habitat is protected from human disturbance. Suitable habitat is defined as 10 x 10 m cells with a probability of nest occurrence above the calculated suitability threshold in blue (0.474); warmer colors (red, yellow, orange) indicate areas with higher suitability. To the south, a large swath of suitable habitat only becomes available to piping plovers under full protection from human disturbance

**Figure 6:**
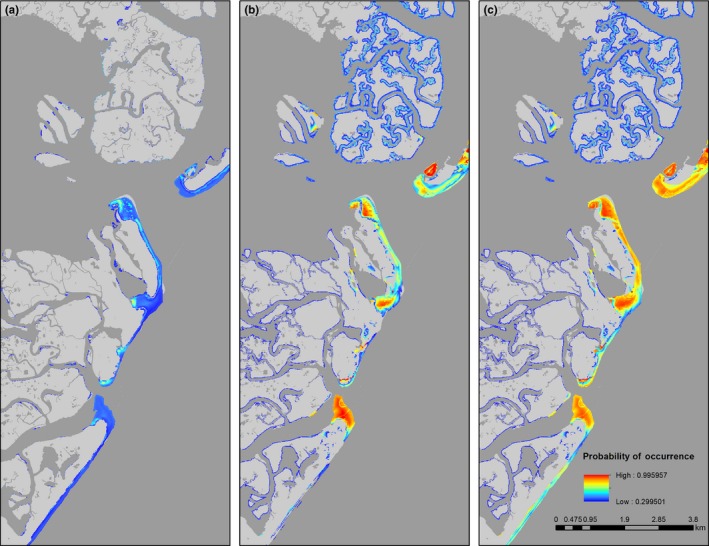
Example map of changes to the location and extent of habitat suitable for nesting by black skimmers (colored areas) on Pullen Island, New Jersey, USA (a) without human disturbance protections, (b) under the current levels of protection, and (c) under a scenario in which all potential habitat is protected from human disturbance. Suitable habitat is defined as 10 x 10 m cells with a probability of nest occurrence above the calculated suitability threshold in blue (0.300); warmer colors (red, yellow, orange) indicate areas with higher suitability. Overall habitat quality (suitability score) improves with human disturbance protections

**Figure 7:**
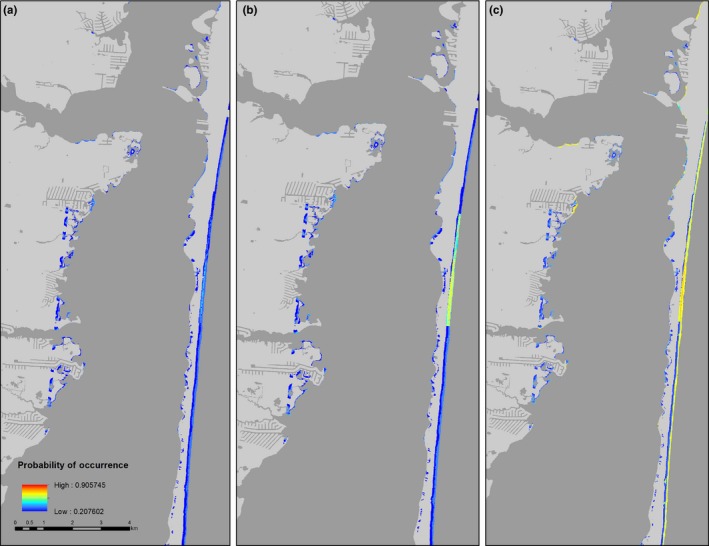
Example map of changes to the location and extent of habitat suitable for nesting by American oystercatchers (colored areas) on Island Beach State Park, New Jersey, USA (a) without human disturbance protections, (b) under the current levels of protection, and (c) under a scenario in which all potential habitat is protected from human disturbance. Suitable habitat is defined as 10 x 10 m cells with a probability of nest occurrence above the calculated suitability threshold in blue (0.208); warmer colors (red, yellow, orange) indicate areas with higher suitability. The quality of nesting habitat considerably improves across a large portion of habitat that exists without human disturbance protections

**Figure 8:**
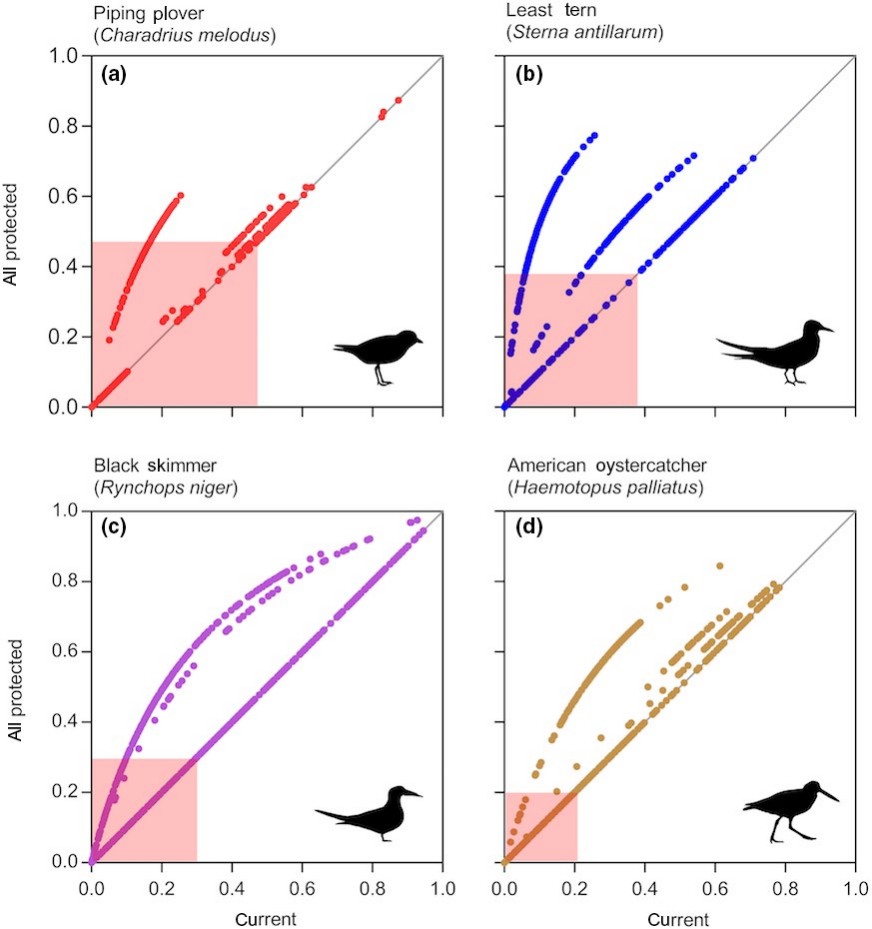
Comparison of current versus future probabilities of nest occurrence for four species of beach‐nesting birds on the New Jersey, US coastline. Future probabilities are modeled under a scenario where all areas are managed to protect birds from impacts caused by human recreational uses of beaches and dunes (i.e., “all protected”). The spatial resolution of the models is 10 x 10 m cells corresponding with points plotted here. The 1:1 line indicates no change in a given cell's suitability score under the all protected scenario. Colored boxes represent the calculated threshold probability of nest occurrence

In general, habitat suitability for all species improved considerably on beaches near estuarine inlets, and on sandy spits that are characterized by broad sandflats backed by dunes (Figures 4, 5, 6, 7). Under the current protection scenario, the suitability scores of some previously unprotected areas increased above the calculated suitability thresholds, adding additional sites to the conservation network. In areas predicted to support nesting regardless of protection status (scores already above the suitability threshold), reducing disturbance improved habitat quality. Under the “all protected” scenario, nesting habitat for piping plovers mainly improved by making currently marginal habitats more suitable for nesting to occur (Figure 7a, Table 2). Least tern habitat suitability changes demonstrated a similar pattern, with many currently unsuitable areas shifting to suitable under the all protected scenario. There was also some increase in least tern habitat quality in areas that already contained nesting birds (Figure 8b; Table 2). Predicted nesting habitat of black skimmers and American oystercatchers improved under a future all protected scenario through enhancement of the quality of existing habitat, and, to a lesser extent, augmenting some new areas (Figure 8c,d; Table 2).

**Table 2:** Change in habitat suitability scores for four beach‐nesting bird species along the coastline of New Jersey, USA. Cell entries are the change in nesting probabilities per 10 x 10 m cell, comparing the current protections scenario with a future scenario of all cells protected from human disturbance (i.e., ∆ Nest P = future P_nest_ – current P_nest_)

Current probability class	Least tern		Piping plover		Black skimmer		American oystercatcher	
	Mean	*SE*	Mean	*SE*	Mean	*SE*	Mean	*SE*
0.10–0.20	0.41	0.01	0.30	0.00	0.02	0.00	0.01	0.00
0.20–0.30	0.19	0.03	0.27	0.01	0.02	0.00	0.08	0.01
0.30–0.40	0.16	0.01	0.02	0.01	0.03	0.00	0.15	0.01
0.40–0.50	0.07	0.02	0.03	0.00	0.07	0.01	0.05	0.01
0.50–0.60	0.02	0.01	0.01	0.00	0.17	0.02	0.06	0.01
>0.60	0.00	na	0.00	0.00	0.02	0.00	0.01	0.00

Under full protection from human disturbance, maximum predicted suitability for all species occurred on sandy substrates fronting low‐energy intertidal zones (bay or inlet shores). When human recreation was present (no protection), suitability scores of these habitats decreased below the calculated threshold, degrading to the point of being unusable for least terns and piping plovers to nest (Figure 4a and 5a). For American oystercatchers and black skimmers, suitability scores remained marginally above the threshold without human disturbance protections (Figures 6 and 7). Areas demonstrating little to no change in suitability, regardless of protection scenario, included low‐elevation marsh islands and narrow ocean or bay beaches backed by dense development.

## 4 DISCUSSION

Human activities on sandy beaches and coastal dunes can substantially modify the distribution, abundance, and fitness of birds (Murchison, Zharikov, & Nol, 2016). It follows that beach and dune habitats differ in quality and suitability for nesting at least partly based on the intensity and frequency of human disturbance and the management interventions targeted to reduce it (Dowling & Weston, 1999). Here, we show that present conservation protections have dramatically increased the potential nesting habitat extent for piping plovers and least terns, two threatened beach‐nesting birds inhabiting a densely populated coastline. In fact, they are highly unlikely to breed in New Jersey without active management of recreational beach use. While current conservation strategies are successfully maintaining local populations, modeling scenarios where protection is extended across the entire study area indicate substantial potential for increasing conservation benefits to these species through additional restrictions and/or management interventions in more areas. Population growth for these species is likely habitat‐limited, based on evidence from recent pulses in reproductive output following significant storm‐induced habitat gains along the New Jersey coastline (Heiser & Davis, 2017). Therefore, additional habitat protections likely are crucial for these federally listed species to achieve population recovery goals (Sidle & Harrison, 1990; USFWS, 1996). Our models predict an additional 1,674 ha and 1,075 ha of suitable nesting habitat for least terns and piping plovers, respectively, across our study area, effectively tripling the current conservation benefit for these species.

We also show that even for broadly ecologically similar species, human disturbance protections will serve some species better than others. The models indicate that in contrast to least terns and piping plovers, American oystercatchers have less future scope for spatial conservation benefits. Therefore, the magnitude of benefit conferred upon a species from human protections is likely dependent on its niche breadth and sensitivity to disturbance. Our models predict extensive potential nesting areas for American oystercatchers (~4,779 ha) regardless of the degree of human disturbance protections, likely because their broad habitat requirements allow them to occupy habitats less frequented by humans. For this species, conservation protections augment available habitat only modestly (~3% at current protection levels and ~6% under total protection); however, their habitat flexibility may, to some degree, allow them to tolerate displacement from high‐use recreational sites (Clemens, Weston, Haslem, Silcocks, & Ferris, 2010; Rödder et al., 2009). For species with more specific habitat requirements (especially during nesting), management of human disturbance is much more important (Schlacher, Weston, Lynn, & Connolly, 2013; Weston et al., 2014). Indeed, human recreational use can significantly degrade habitat quality (Maslo, Leu, et al., 2016; Schlacher et al., 2015), even for more tolerant species, and negatively impact reproductive output (Schlacher, Carracher, et al., 2016). Therefore, extending human disturbance protections will also likely confer a significant conservation benefit by improving nest success and chick survival when birds breed in protected areas, ultimately increasing population growth (Cohen et al., 2016; McGowan et al., 2005).

The comparative models illustrated here represent an important foundational step in establishing a successful strategy to improve conservation returns for these beach‐nesting birds. At a minimum, they identify locations that are not likely to change in suitability once protected from human disturbance (Figure 8). Areas experiencing no change in habitat suitability under different protection scenarios may be sites that are geomorphologically inconsistent with species’ habitat needs; these areas may be prime locations in which to promote public recreation. More importantly, the models highlight locations that are likely to transition from unsuitable to suitable if human disturbance is mitigated. Increases may result from the re‐emergence of microhabitat due to features important in nest‐site selection (incipient dune formation, shell cover, etc.), which are prevented by beach‐raking and trampling by vehicles and pedestrians (Kelly, 2016; Priskin, 2003; Šilc, Caković, Küzmič, & Stešević, 2017). Such activities also reduce the abundance of prey resources (Schlacher et al., 2017; Schlacher, Carracher, et al., 2016). Removing these stressors may reestablish these coastal processes, thereby increasing habitat quality. Alternatively, existing geomorphic conditions may be marginally suitable for nesting, but human presence prevents birds from attempting to establish breeding territories (Ciuti et al., 2012).

A test of this hypothesis would be to experimentally close or vary the management of humans in areas with modeled scores just below the suitability threshold and monitor bird activity. However, manipulative experiments could be costly and might result in negative effects if not implemented carefully (create sink habitats). In addition, they would require a surplus of breeding individuals. Population size of these threatened species is typically low; therefore, there may not be enough birds to occupy all new habitat areas. Beach‐nesting birds also have high site fidelity and typically return to the sites in which they have previously nested (Cohen, Fraser, & Catlin, 2006). Therefore, protected sites without a history of nesting may not be immediately occupied. Post‐breeding individuals and juveniles prospect new nesting sites after the breeding season in late summer/early fall (Davis et al., 2017; Faaborg et al., 2010), making it more probable that occupancy by target species may not occur until at least two seasons following intervention. Changes in the condition of other habitat areas will likely play an important role in determining occupancy of newly protected and/or intensively managed areas.

## 5 CONCLUSIONS

To make informed decisions about where and how to invest conservation funds, a critically important factor is to assess data on the capacity of the landscape to accommodate additional, high‐quality habitat (Lindenmayer et al., 2008; Roberts et al., 2003). In this context, we provide estimates of the total area of habitat that is predicted to accommodate breeding under a scenario where all potential habitat is managed to reduce detrimental impacts from recreational beach use. Species distribution models can reliably predict the amount of potential habitat that is present across a study area, but they cannot measure habitat quality as defined by increasing population viability. Solidifying the link between habitat suitability and demographic rates (in our case, egg and juvenile survival) would further refine the ability of conservation managers to decide between alternative management strategies to optimize return on investment. What is needed are spatially explicit data on demographic rates to make good decisions on conservation investments. Equally, understanding human recreational site selection is of importance. Knowledge of what factors influence human choice of beaches could inform management decisions on which sites within a region to protect in ways that will maximize conservation outcomes while also maintaining recreational opportunities. Similarly, linking habitat quality with specific management interventions may provide further guidance on what recreational activities might be allowable near nesting areas, potentially facilitating coexistence of these seemingly conflicting priorities. Thus, the data provided here represent the first fundamental step to enhance spatial management of bird habitats on ocean beaches and coastal dunes.

## AUTHOR CONTRIBUTIONS

BM and TP conceived of the ideas and designed the methodology. BM, KL, and TP acquired the data sets, and BM and KL performed all habitat modeling. BM and TS performed the statistical analyses. All authors contributed to manuscript drafts.

## DATA ACCESSIBILITY

Per a contractual agreement with the funder, upon publication all data will be publicly available at the United States Fish and Wildlife Service North Atlantic Landscape Conservation Cooperative website, under the *Coastal Resiliency: Marshes, Beaches and Aquatic Systems* project page: https://northatlanticlcc.org/teams/coastal-resiliency/projects/hurricane-sandy/rutgersbeachmodeling.
